# Coverage, spatial distribution and determinants of childhood inactivated poliovirus vaccine immunization in Ethiopia

**DOI:** 10.1371/journal.pone.0301933

**Published:** 2024-05-31

**Authors:** Tadesse Tarik Tamir, Belayneh Shetie Workneh, Enyew Getaneh Mekonen, Alebachew Ferede Zegeye

**Affiliations:** 1 Department of Pediatrics and Child Health Nursing, School of Nursing, College of Medicine and Health Sciences, University of Gondar, Gondar, Ethiopia; 2 Department of Emergency and Critical Care Nursing, School of Nursing, College of Medicine and Health Sciences, University of Gondar, Gondar, Ethiopia; 3 Department of Surgical Nursing, School of Nursing, College of Medicine and Health Sciences, University of Gondar, Gondar, Ethiopia; 4 Department of Medical Nursing, School of Nursing, College of Medicine and Health Sciences University of Gondar, Gondar, Ethiopia; University of Arizona, College of Medicine-Phoenix, UNITED STATES

## Abstract

**Introduction:**

Polio eradication is a current and common strategy throughout the globe. The study of the newly introduced inactivated poliovirus vaccine provides a grasp on the current status of immunization and identifies any disparities in the implementation of the vaccine throughout Ethiopia. Thus, this study aimed to demonstrate the spatial distribution, coverage, and determinants of inactivated poliovirus vaccine immunization in Ethiopia.

**Method:**

Spatial distribution and determinants of inactivated poliovirus vaccine immunization in Ethiopia were conducted using Ethiopian mini-demographic and health survey 2019 data. A total of 2,056 weighted children aged 12 to 35 months were included in the analysis. The association between the outcome and explanatory variables was determined by commuting the adjusted odds ratio at a 95% confidence interval. The p-value of less than 0.05 was used to declare factors as significantly associated with the inactivated poliovirus vaccine immunization.

**Result:**

The weighted national coverage of inactivated poliovirus vaccine immunization in Ethiopia was 51.58% at a 95% confidence interval (49.42, 53.74). While the rates of inactivated poliovirus vaccine immunization were observed to be greater in Addis Ababa, Tigiray, Amahara, and Benishangul Gumuz provinces and lower in the Somali, Afar, and SNNPR provinces of Ethiopia, Antenatal care follow-up, place of delivery, place of residence, and region were significantly associated with inactivated poliovirus immunization in Ethiopia.

**Conclusion:**

The distribution of inactivated poliovirus immunization was spatially variable across Ethiopia. Only about half of the children aged twelve to thirty-five months received the inactivated poliovirus vaccine in the country. The factors, both at the individual and community level, were significantly associated with inactivated poliovirus immunization. Therefore, policies and strategies could benefit from considering antenatal care follow-up, place of delivery, place of residence, and region while implementing inactivated poliovirus vaccine immunization.

## Introduction

Poliomyelitis is an acute viral disease that is mostly contagious and neurotropic, caused by serotypes 1, 2, and 3 of the poliovirus (PV) [1]. Poliovirus infection causes a wide range of clinical manifestations, from nonspecific febrile illnesses to aseptic meningitis, paralytic paralysis, and death [1]. Serology is used to diagnose the poliomyelitis and there is no specific treatment for poliovirus [2]. Poliomyelitis can be prevented with immunization. There are two types of polio vaccinations: Salk-type (inactivated polio vaccine, IPV) and Sabin-type (live attenuated oral poliovirus vaccine (OPV) (2). Universal vaccination of infants and children is the only means of establishing and maintaining population immunity against poliovirus to prevent poliomyelitis cases and epidemics [1, 3]. Polio eradication has been a major global health objective for the last two decades [4].

Globally, the number of wild polio cases found declined from 256 to 171 between 2013 and 2014, and then to 33 recorded cases in 2018 [5]. Yet the disease is reviving in several countries around the world. According to the WHO, the disease has recently been found in four WHO areas (African, Eastern Mediterranean, Southeast Asia, and Western Pacific) [6]. The Global Polio Eradication Initiative (GPEI) has sketched out an endgame strategy to eradicate poliomyelitis, which involves moving from the oral poliovirus vaccine (OPV) to the inactivated poliovirus vaccine (IPV) [3]. To meet this goal, the WHO advised that all countries incorporate at least one dose of IPV into routine immunization campaigns by the end of 2015 [3]. In the same year (2015) of WHO recommendation, Ethiopia introduced the inactivated poliovirus vaccine into the national immunization schedule. The IPV is provided at 14 weeks, along with the third dose of the oral poliovirus vaccine.

The final report of the 2019 Ethiopian mini-demographic and health survey (2019 EMDHS) implicated the existence of disparities in childhood vaccination in terms of residency, region, household wealth index, and maternal educational status [7]. However, IPV immunization status was not reported by the 2019 EMDHS. Hence, this study aimed to demonstrate the disparity by determining national and regional coverage, spatial distribution, and determinants of IPV immunization in Ethiopia using data from the 2019 EMDHS, the first demographic and health survey in Ethiopia after the inactivated polio virus vaccine was introduced into the immunization schedule of the country. The findings of the study will help point out and address the disparities in the newly introduced IPV immunization throughout the country. In addition, a grasp of the vaccination status of IPV across regions of Ethiopia can be an input for further implementation of the vaccine.

## Methods

### Data source and study setting

The coverage, spatial distribution and determinants of childhood inactivated poliovirus vaccine (IPV) immunization was done using secondary analysis of Ethiopian mini demographic and health survey (EMDHS) 2019 dataset. The data were accessed from the Monitoring and Evaluation to Assess and Use Results Demographic and Health Survey (MEASURE DHS) program through a formal request and are available at https://www.dhsprogram.com/data/available-datasets.cfm. Registration is the sole requirement for access to the dataset. The study was conducted in Ethiopia; an East African country. As it was accessed from the source shape file (https://africaopendata.org/dataset/ethiopia-shapefiles), there are eleven provinces in Ethiopia, namely, Tigray, Afar, Amhara, Oromia, Somali, Benishangul, SNNPR, Gambela, Harari, Addis Ababa and Dire Dawa.

#### Population and sampling

The source population of the study were all children aged 12 to 35 months in Ethiopia, whereas all children 12 to 35 months in the selected enumeration areas (EAs) or sampling clusters were the study population.

The EMDHS used a two-stage stratified sampling technique. In the first stage, 305 clusters/enumeration areas were randomly selected with a probability sampling stratified with urban and rural areas. In the second stage, a fixed number of 30 households per cluster were selected using probability sampling. Mothers of all under 35 months of age children in each selected household were asked for vaccination status of their children. A total sample of 2,056 weighted children aged 12 to 35 months was included in the analysis of this study.

### Variables and measurement

The outcome variable of the study was inactivated poliovirus vaccine immunization. It was dichotomized as “yes” for children who received IPV and “no” for those who did not receive the vaccine. Since the DHS data is hierarchical, two levels of independent variables were included in this study. Firstly, individual level variables such as maternal age, maternal educational status, household wealth status, ANC visits, place of delivery and birth order were included. Secondly, community level variables such as residence, region, community women literacy level, community poverty level and community ANC utilization.

The community-level factors (community women’s literacy level, community poverty level, and community ANC usage) were generated by integrating the individual-level factors with the cluster number (v001). To avoid linearity, the proportion values of the community-level variables were dichotomized as high if above the average and low if below the average. The variables in this study were categorized and coded (recoded) based on analytical framework used by EMDHS 2019.

This study used different methods to address its specific objectives. A summary of the study objectives and method of analyses are presented in the table below (Table 1).

**Table 1:** Study objective and respective method of analysis applied by the study.

Study objectives	Method of analysis applied
I. Coverage of childhood inactivated poliovirus vaccine immunization in Ethiopia	Descriptive cross- sectional
II. Spatial distribution of childhood inactivated poliovirus vaccine immunization in Ethiopia	Spatial autocorrelation, SaTScan analysis, hot spot analysis and Kriging interpolation.
III. Determinants of childhood inactivated poliovirus vaccine immunization in Ethiopia.	Multilevel mixed effect

#### Data extraction and management

The children’s Recode (KR) datasets in Stata format were extracted from the MEASURE DHS repository for analysis. Following data extraction, variables were cleaned, recoded and weighted. The STATA version 17 was used for analysis of the coverage and determinants of IPV immunization. Data for mapping IPV immunization were managed (prepared) using STATA 17 and Microsoft excel 2013. The ArcGIS version 10.8 was used for mapping IPV immunization in Ethiopia. The data were weighted using the following variables to aid in drawing accurate conclusions: sample weight (v005), primary sampling unit (v021), and stratum (v023). The weighted prevalence and frequencies of the analysis results were reported using tables and bar charts.

#### Spatial autocorrelation

The spatial dependency of IPV immunization in Ethiopia was determined using spatial autocorrelation (Global Moran’s I). Global Moran’s I statistics have a value between -1 and 1. A Moran’s I value of 0 suggests that the data are randomly distributed, whereas a value close to -1 indicates that the distribution is scattered, and a value close to 1 indicates that the distribution is clustered. A Moran’s I that is statistically significant (P < 0.05) implies that spatial dependence is evident [8].

#### Optimized hot spot analysis

This study used an optimized hot spot analysis technique to identify hot and cold spot areas of IPV immunization in Ethiopia. The Optimized Hot Spot Analysis (Getis-Ord Gi* statistic) tool is an extension of the Hot Spot Analysis (Getis-Ord Gi)*. It aims to identify statistically significant hot and cold spots in your data [9]. It also adjusts results for multiple testing and spatial dependence using the False Discovery Rate (FDR) correction method. The Getis-Ord Gi* statistic quantifies spatial clustering by examining the distribution of features and their neighboring features [9, 10].

#### SaTScan analysis

The spatial SaTScan statistics, which uses a circular scanning window that moves across the study area was used to determine significant clusters of the IPV immunization. Cases, controls, and geographic coordinate data were fitted to the Bernoulli model. For each potential cluster, Log Likelihood Ratio (LLR), Relative Risk (RR) and P-values were used to determine whether the number of observed cases within the potential cluster was significantly higher than expected or not.

#### Spatial interpolation

The spatial prediction of IPV immunization in an unsampled areas of Ethiopia was done based on observed data from sampled areas using a Kriging interpolation. Kriging is an interpolation technique that uses observations from neighboring places to estimate a variable’s value in unsampled locations [11–13]. Kriging reduces the error of the predicted values and expresses the geographic variation using a variogram [11, 13]. Kriging is named after Danie Krige and has its roots in the realm of mining geology [12, 13].

### Multilevel mixed effect analysis

The nature of variables in demographic and health survey data is hierarchical in to two levels. The first hierarchy contains variables within the cluster and the second hierarchy contains variables among clusters. This character of the EMDHS data lead us to use multilevel or mixed effect logistic regression instead of ordinary logistic regression. There are four levels of models in multilevel logistic regression; null model (model 0), model I, model II and model III. Null model-a model without exposure variables, was used to determine variability of IPV immunization among clusters. In model I, the association of individual level factors with IPV immunization was assessed and in model II, the association of community level factors with outcome variable was assessed. The final model (model III) was fitted to assess the association of mixed (individual and community) factors with IPV immunization. The regression equation for multilevel mixed effect model is given as follows [14];

$$\text{log}\left(\frac{\Pi ij}{1-\Pi ij}\right)=\beta o+\beta 1x1ij+\cdots +\beta nxnij+uoj+eij$$

Where; πij is the probability of being immunized for inactivated poliovirus vaccine, (1-πij) is the probability of not being immunized for inactivated poliovirus vaccine, βo is log odds of the intercept, β1,… βn are effect sizes of individual and community-level factors, x1ij… xnij are independent variables of individuals and communities. The quantities uoj and eij are random errors at cluster levels and individual levels, respectively.

The median odds ratio (MOR), intra-cluster correlation coefficient (ICC), and proportional change in variance (PCV) were used to estimate measure of variation (random effect). The MOR quantifies unexplained cluster heterogeneity. When two enumeration areas (clusters) are chosen at random, it is the median value of the odds ratio between a cluster with high odds of IPV immunization and a cluster with low odds of IPV immunization. The ICC measures the variation in IPV immunization across clusters. The PCV is a measure of the total variation in IPV immunization as a result of individual and/or community-level variables.

The parameters-MOR, ICC and PCV are equated as follows [15]; $MOR=\;e^{0.95\sqrt{VC}}\;,ICC=\frac{VC}{VC+3.29}\times 100\%$ and $PCV=\frac{Vnull-VC}{Vnull}\times 100\%$, where; VC = variance of the cluster for respective model and Vnull = variance of the null model.

Model selection was assessed using log likelihood and by computing deviance. The deviance is equal to -2(log likelihood).

The measure of association (fixed effect) was used to estimate the association of individual and community level explanatory variables with IPV immunization. It was assessed using adjusted odds ratio (AOR) and 95% confidence intervals with a p-value of < 0.05.

#### Ethical consideration

This study was based on an analysis of existing survey data in the public domain that is freely available online with all the identifier information anonymized; no ethical approval was required. The first author obtained authorization for the download and usage of the 2019 EMDHS data from MEASURE DHS.

## Results

### Socio-demographic and economic factors of study subjects

A total of 2,056 (1,009 males and 1,047 females) weighted children aged 12 to 35 months were included in the analysis. About half (50.60%) of the children were born to mothers who have no formal education. The majority (81.18%) of children were born to mothers were between 20 and 35 years old. A bit more than half (50.21%) of the study subjects were born at a health facility, and 72.71% of the participants were rural residents (Table 2).

**Table 2:** Socio-demographic and economic factors of study participants.

Variables	Response	Frequency (n)	Percent (%)
Sex of the child	Male	1,009	49.08%
	Female	1,047	50.92%
Maternal Education	No formal education	1040	50.60%
	Primary	755	36.74%
	Secondary and above	260	12.66%
Maternal age	15–19	106	5.17%
	20–35	1669	81.18%
	36–49	281	13.65%
Wealth index	Poor	894	43.51%
	Middle	391	19.00%
	Rich	771	37.49%
ANC visits	No visit	456	25.38%
	Had visit	1340	74.62%
Place of delivery	Home	1,023	49.79%
	Health facility	1,032	50.21%
Birth order	First	462	22.48%
	Second	378	18.38%
	Third	284	13.84%
	Fourth and above	931	45.30%
Residence	Urban	561	27.29%
	Rural	1494	72.71%
Region	Tigray	138	6.72%
	Afar	32	1.58%
	Amhara	410	19.94%
	Oromia	834	40.58%
	Somali	129.89	6.32%
	Benishangul	24	1.16%
	SNNPR	395	19.23%
	Gambela	8	0.39%
	Harari	6	0.30%
	Addis Ababa	66	3.22%
	Dire Dawa	11	0.55%
Community women literacy level	Low	932	45.37%
	High	1123	54.63%
Community poverty level	Low	1,228	59.72%
	High	828	40.28%
Community ANC Utilization	Low	857	41.71%
	High	1198	58.29%

ANC: antenatal care, SNNPR: Southern Nations Nationalities and Peoples Region.

### Coverage of inactivated poliovirus vaccine immunization in Ethiopia

**Fig 1:**
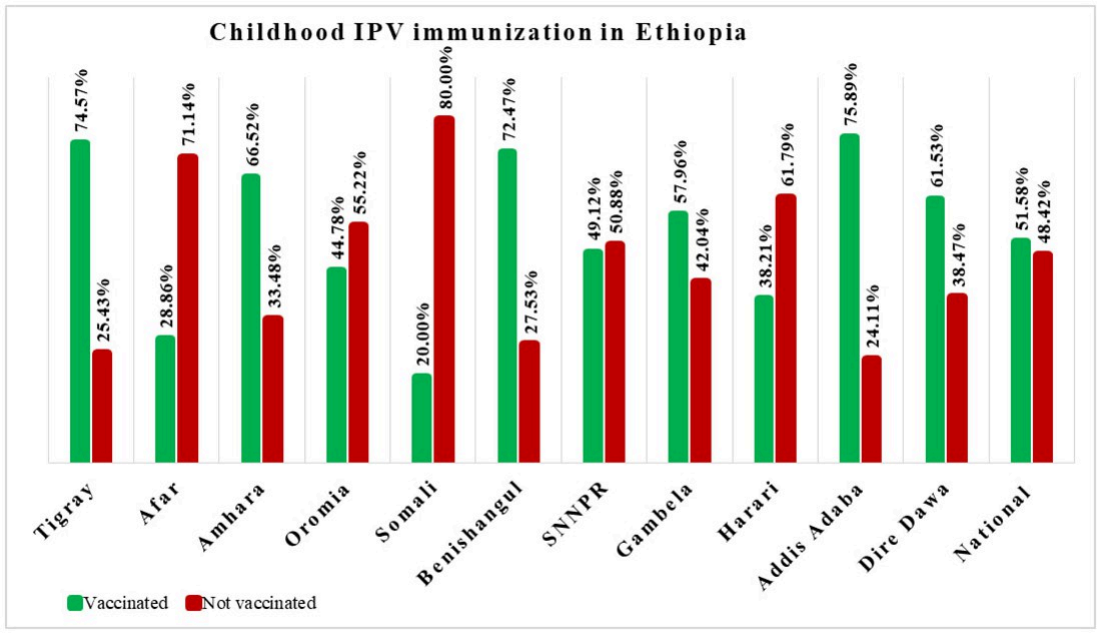
Regional and national coverage of childhood inactivated poliovirus vaccine immunization in Ethiopia; IPV: Inactivated Poliovirus Vaccine, SNNPR: Southern Nations Nationalities and Peoples Region.

The weighted national coverage of inactivated poliovirus vaccine immunization in Ethiopia was 51.58% at a 95% confidence interval (49.42, 53.74). By place of residence, the coverage of inactivated polio vaccine immunization was 68.67% and 45.17% in urban and rural areas of Ethiopia, respectively. Low coverage (20.00%) was observed in the Somali region and high coverage (75.89%) in Addis Ababa (Fig 1).

### Spatial autocorrelation childhood inactivated polio vaccine immunization in Ethiopia

**Fig 2:**
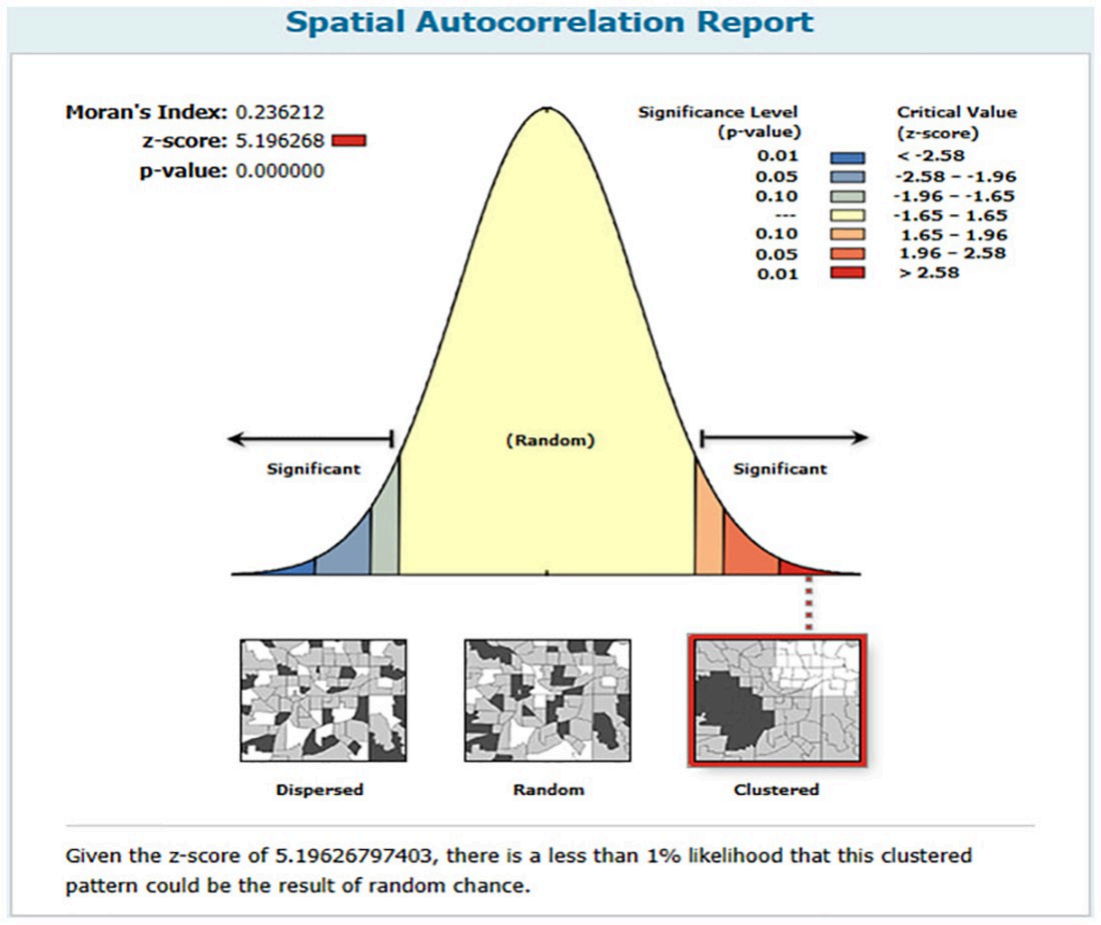
Spatial autocorrelation analysis of inactivated poliovirus vaccine immunization in Ethiopia.

The result of the spatial autocorrelation of this study show that the distribution of inactivated poliovirus vaccine immunization varied significantly across Ethiopia (Moran’s Index: 0.236212, Z-score: 5.196268, p-value<0.0001) (Fig 2). This suggests that there was a spatial variation in inactivated poliovirus vaccine immunization in Ethiopia.

### Hotspot analysis of childhood inactivated poliovirus vaccine immunization in Ethiopia

**Fig 3:**
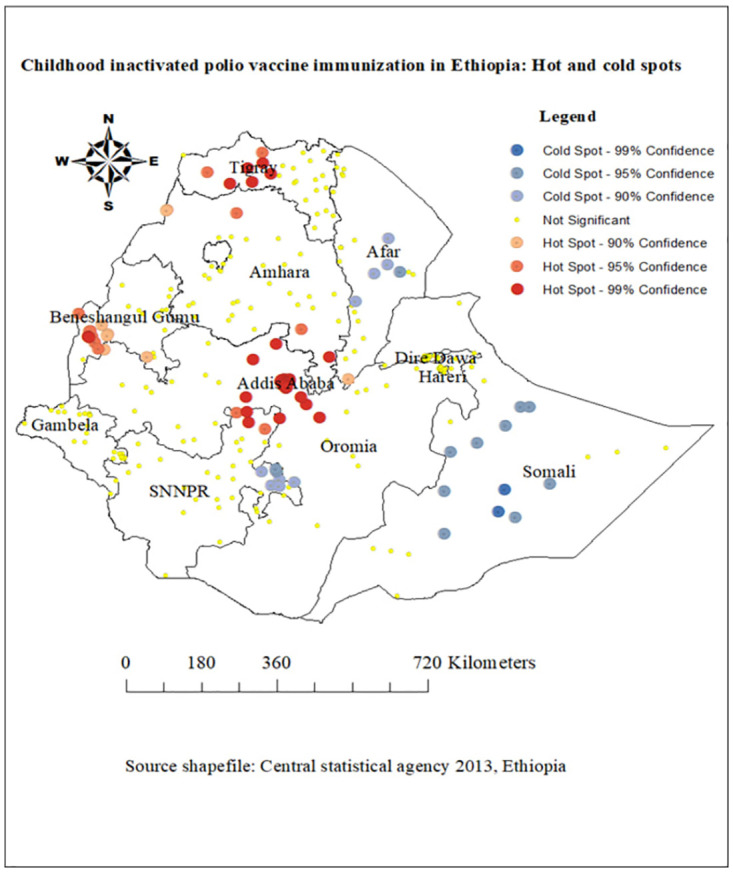
Optimized hotspot analysis of childhood inactivated poliovirus vaccine immunization in Ethiopia, source shape file: https://africaopendata.org/dataset/ethiopia-shapefiles, map output is the result of our own analysis and the figure is not identical to the original image and is therefore for illustrative purposes only.

Optimized hot spot analysis was used to determine hot and cold spots for childhood inactivated poliovirus vaccine immunization in Ethiopia. It was determined that the optimal fixed distance between a cluster and at least one neighboring cluster was 152917.5 meters. Following optimized hot spot analysis, there were eighty five output features (spots) statistically significant based on false discovery rate (FDR) correction for multiple testing and spatial dependence. About 7.9% of features had less than 8 neighbors based on distance band of 152917.5 meters. Accordingly, red output features represent hot spots where high inactivated poliovirus vaccine immunization values cluster and blue output features represent cold spots where low inactivated poliovirus vaccine immunization values cluster. While Addis Ababa, Tigiray, some part of Amahara, and Beninshangul Gumuz provinces were hot spot areas of inactivated polio vaccine immunization, Somali, Afar and SNNPR were cold spot areas of inactivated poliovirus vaccine immunization (Fig 3).

### SaTScan analysis of childhood inactivated polio vaccine immunization in Ethiopia

**Fig 4:**
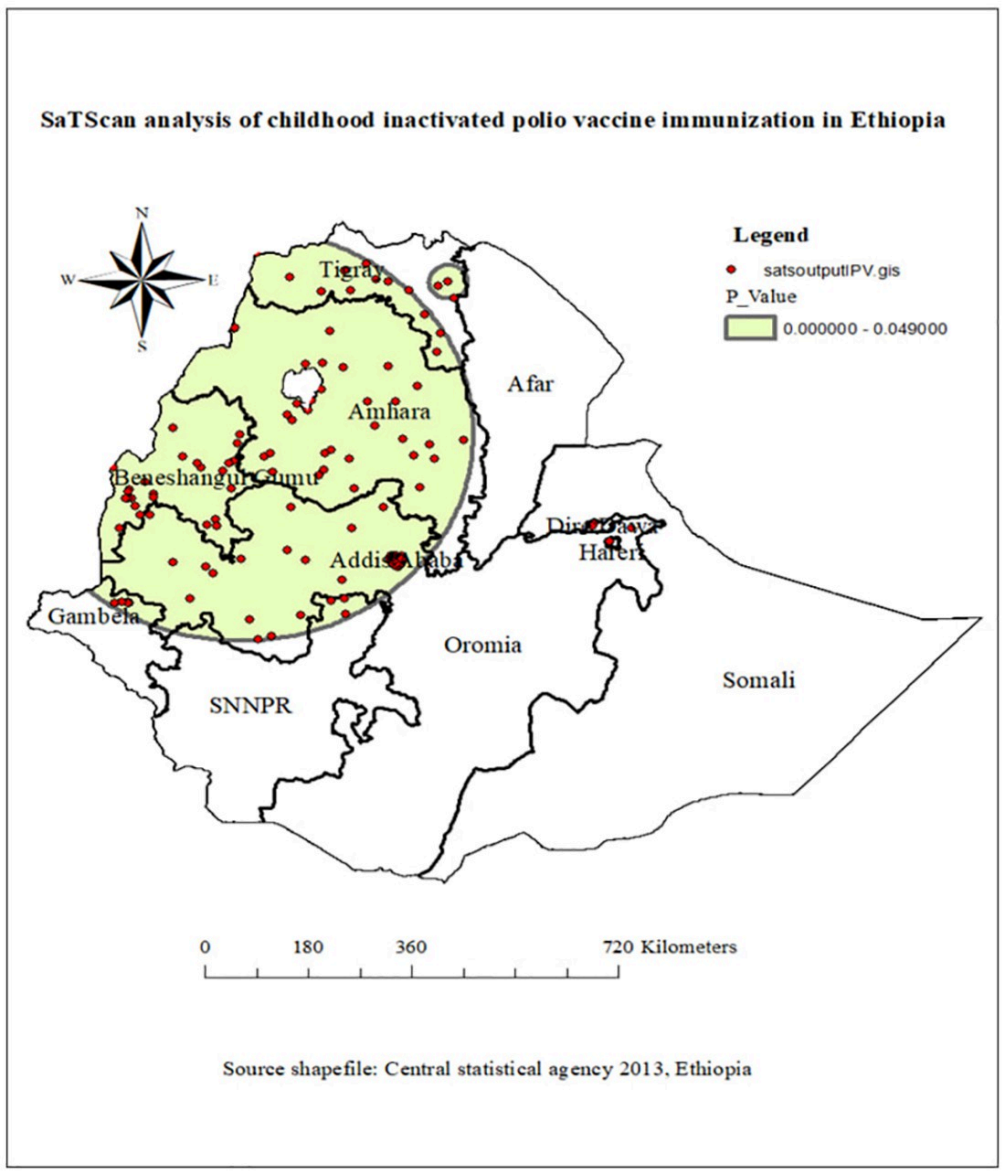
SaTScan analysis of childhood inactivated poliovirus vaccine immunization in Ethiopia, source shape file: https://africaopendata.org/dataset/ethiopia-shapefiles, map output is the result of our own analysis and the figure is not identical to the original image and is therefore for illustrative purposes only.

The SaTScan analysis identified a total of 222 significant clusters. The 119 of total identified significant clusters were primary clusters. The primary clusters were located at 11.166027 North and 36.342701 East, with a 397.04km radius. Children living in the primary cluster were 64% more likely to immunized for inactivated poliovirus vaccine compared to children outside the cluster window (relative risk = 1.64 and log-likelihood Ratio = 71.84, P-value<0.0001) (Table 3). The most likely clusters of childhood inactivated poliovirus vaccine immunizations were observed predominantly in some parts of Tigray, Amhara, Benishangul Gumuz, Addis Ababa, Dire Dawa, Harari, and parts of the Oromia region (Fig 4).

**Table 3:** The SaTScan analysis of childhood inactivated poliovirus vaccine immunization in Ethiopia.

Cluster	Enumeration areas (cluster) detected	Coordinates/radius	Population(n)	Cases(n)	RR	LLR	P-Value
Primary	165, 162, 163, 80, 148, 79, 166, 75, 53, 77, 161, 158, 164, 160, 74, 159, 54, 81, 59, 76, 52, 70, 72, 57, 119, 167, 84, 168, 169, 149, 150, 156, 71, 82, 85, 55, 83, 157, 86, 73, 151, 153, 58, 146, 154, 60, 152, 155, 147, 98, 93, 56, 92, 99, 87, 120, 61, 118, 170, 65, 100, 78, 51, 22, 21, 62, 67, 63, 94, 112, 66, 9, 262, 4, 259, 257, 258, 256, 261, 260, 265, 263, 266, 174, 97, 276, 267, 264, 275, 8, 273, 270, 269, 277, 268, 274, 95, 171, 271, 279, 272, 18, 278, 280, 207, 7, 208, 209, 64, 211, 13, 23, 1, 230, 176, 20, 91, 96, and 2.	(11.166027 N, 36.342701 E) / 397.04 km	1108	622	1.64	71.84	<0.0001
Secondary	3, 17, and 25.	(13.801030N, 39.592869 E)/32.56 km	39	30	1.86	10.13	0.014
Tertiary	283, 284, 285, 282, 291, 288, 297, and 286.	(9.608872 N, 41.844131 E) / 2.69 km	67	44	1.59	7.94	0.073
Quaternary	108, 305, 304, 303, 302, 296, 281, 287, 286, 288, 284, 294, 282, 292, 283, 285, 291, 295, 293, 297, 290, 289, 231, 301, 232, 246, 233, 298, 237, 243, 236, 244, 300, 234, 235	(9.312527 N, 41.610359 E) / 56.55 km	51	32	1.52	4.62	0.755
5	109	(9.538250 N, 42.452740 E) / 0 km	13	10	1.85	3.35	0.99

### Kriging interpolation of childhood inactivated poliovirus vaccine immunization in Ethiopia

**Fig 5:**
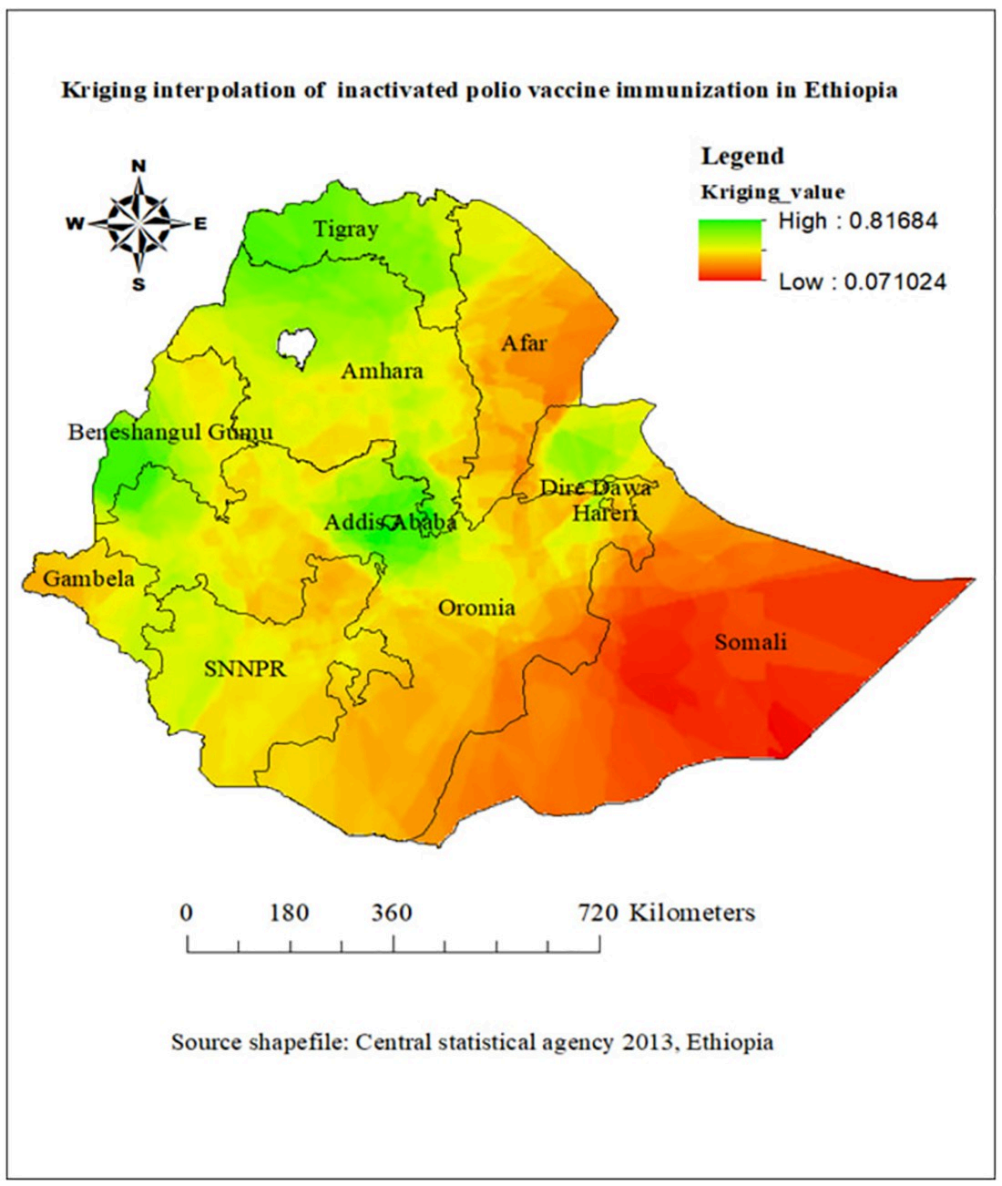
Kriging interpolation of childhood inactivated poliovirus vaccine immunization in Ethiopia, source shape file: https://africaopendata.org/dataset/ethiopia-shapefiles, map output is the result of our own analysis and the figure is not identical to the original image and is therefore for illustrative purposes only.

The output of Kriging interpolation analysis (Fig 5) shows predicted locations for high and low inactivated polio vaccine immunization values throughout Ethiopia. The green colors on the map show areas predicted to have high inactivated polio vaccine immunization and red colors on the map show areas predicted to have low inactivated poliovirus vaccine immunization. Accordingly, Addis Ababa, Amhara, Tigray, Benishangul Gumuz, Dire Dawa, East of Gambela, and Western border f Oromia were regions were predicted to have higher IPV rates of immunization. Conversely, Afar, Somali, SNNPR, Oromia and some portion of Gambela were regions with lower prediction of IPV immunization in Ethiopia (Fig 5).

### Measures of variation (Random effect) and model fit statistics

The ICC = 0.27 (0.22, 0.33) in the null model (model 0) shows that the variation between enumeration areas (clusters) was responsible for 27% of total variation in inactivated poliovirus vaccine immunization in Ethiopia. The unexplained heterogeneity in inactivated polio vaccine immunization between enumeration areas was high (MOR = 2.88) without inclusion of factors. The unexplained heterogeneity was reduced to 1.78 in the final model when both individual and community factors were added to the outcome. The MOR = 1.78 in Model III means if we pick a child from two separate clusters selected randomly, the odds of inactivated poliovirus vaccine immunization are 1.78 times higher for a child from a cluster with a high probability of IPV immunization compared to a child from a cluster with a low probability. When compared to null model, 50% and 66.94% of the total variation in IPV immunization was attributed to individual and community level factors, respectively. In addition, about 70% of overall variation in IPV immunization was attributed to both individual and community level factors (PCV = 70.16%).

The final model (model III), the model with the highest log likelihood ratio (LLR) and lowest value of deviance, was the best fit to identify factors associated with childhood IPV immunization (Table 4).

**Table 4:** Random effect and model fit statistics of childhood inactive polio vaccine immunization in Ethiopia.

Parameters	Model 0	Model I	Model II	Mode III
**Random effect**				
Variance	1.24	0.61	0.41	0.37
ICC	0.27 (0.22, 0.33)	0.16 (0.12, 0.21)	0.11 (0.08, 0.16)	0.10 (0.07, 0.15)
MOR	2.88	2.10	1.84	1.78
PCV	Reference	50.00%	66.94%	70.16%
**Model fit Statistics**				
LL	-2018.20	-1742.50	-1926.77	-1706.00
Deviance	4036.40	3485.00	3853.54	3412.00

ICC: Intra-cluster Correlation Coefficient, MOR: Median Odds Ratio, LL: Log Likelihood and PCV: Proportional Change in Variation.

### Measures of association (fixed effect)

Following multilevel (mixed effect) logistic regression analysis of this study, two individual level factors (ANC visit and place of delivery) and two community level factors (residence and region) were significantly associated with childhood inactivated poliovirus vaccine immunization.

Regarding the individual level factors, the odds of IPV vaccination were 2.41 times higher among children born to mothers who had ANC visits compared to children born to mothers who had no visits (AOR = 2.41, 95% CI: 1.87, 3.11). In addition, the odds of IPV immunization were 1.29 times higher among children born at health facility compared to those born at home (AOR = 1.29, 95% CI: 1.03, 1.61).

With respect to community level factors, when compared to children who live in rural areas of Ethiopia, the odds of IPV immunization were 1.62 times higher among children who live in urban areas (AOR = 1.62, 95% CI: 1.15, 2.30). Moreover, taking Somali as reference, the odds of IPV immunization were 3.94, 2.89, 1.13 and 3.21 times higher in Tigray, Amhara, Addis Ababa and Benisgangul regions, respectively (Table 5).

**Table 5:** Multilevel analysis of determinants of childhood inactivated polio vaccine immunization in Ethiopia.

Individual and community level factors		Model I	Model II	Model III
		AOR (95% CI)	AOR (95% CI)	AOR (95% CI)
Maternal Education	No formal education	1		1
	Primary	1.04 (0.84, 1.29)		1.01 (0.80, 1.26)
	Secondary and above	1.24 (0.91, 1.69)		1.12 (0.81, 1.53)
Maternal age	15–19	0.67 (0.45, 0.98)		0.72 (0.49, 1.05)
	20–35	1		1
	36–49	1.01 (0.76, 1.35)		0.91 (0.69, 1.22)
Wealth index	Poor	0.73 (0.56, 0.96)		0.82 (0.62, 1.09)
	Middle	1		1
	Rich	1.36 (1.02, 1.82)		1.23 (0.90, 1.67)
ANC visit	No visit	1		1
	Had visit	2.76 (2.15, 3.54)		2.41 (1.87, 3.11)*
Place of delivery	Home	1		1
	Health facility	1.46 (1.17, 1.82)		1.29 (1.03, 1.61)*
Birth order	First	1		1
	Second	0.93 (0.70, 1.22)		0.97 (0.73, 1.28)
	Third	1.07 (0.79, 1.46)		1.10 (0.81, 1.49)
	Fourth and above	1.05 (0.79, 1.39)		1.13 (0.85, 1.49)
Residence	Urban		1.93 (1.38, 2.69)	1.62 (1.15, 2.30)*
	Rural		1	1
Region	Tigray		6.19 (3.28, 11.66)	3.94 (2.06, 7.55)*
	Afar		1.09 (0.62, 1.93)	0.87 (0.48, 1.57)
	Amhara		4.23 (2.38, 7.50)	2.89 (1.60, 5.21)*
	Oromia		2.21 (1.26, 3.87)	1.62 (0.91, 2.89)
	Somali		1	1
	Benishangul		4.91 (2.71, 8.92)	3.21 (1.74, 5.92)*
	SNNPR		2.22 (1.26, 3.88)	1.57 (0.88, 2.80)
	Gambela		2.48 (1.35, 4.58)	1.75 (0.93, 3.27)
	Harari		1.64 (0.89, 3.04)	0.99 (0.53, 1.89)
	Addis Ababa		4.82 (2.41, 9.66)	1.13 (1.53, 6.35)*
	Dire Dawa		2.57 (1.40, 4.71)	0.49 (0.81, 2.84)
Community women literacy level	Low		1	1
	High		1.15 (0.87, 1.51)	1.09 (0.81, 1.45)
Community poverty level	Low		1.38 (1.04, 1.82)	1.02 (0.74, 1.39)
	High		1	1
Community ANC Utilization	Low		1	1
	High		1.43 (1.07, 1.91)	1.02 (0.75, 1.39)

AOR: Adjusted Odds Ratio, ANC: Antenatal Care, CI: Confidence interval, SNNPR: Southern Nations Nationalities and People’s Region and *: Statistically significant (P-value<0.05).

## Discussion

The 2019 Ethiopian mini-demographic and health survey (EMDHS 2019) final report implicated the existence of disparities in childhood vaccination in terms of residency, region, household wealth index, and maternal educational status [7]. However, IPV immunization status was not reported by the EMDHS 2019. Hence, this study disclosed coverage, spatial distribution, and determinants of IPV immunization in Ethiopia.

The weighted national coverage of inactivated poliovirus vaccine immunization in Ethiopia was 51.58% at a 95% CI (49.42, 53.74). The IPV immunization coverage in this study was far below the government target of Health Sector Transformation Plan IV (HSTP) (95%) [16]. The coverage in this study was also lower than coverage in a study in Uganda, 71% [17]. The lower coverage of IPV immunization in Ethiopia might be due to the fact that the country has been facing internal instabilities and population displacement since the vaccine was introduced, which might have prevented mothers from vaccinating their children. Moreover, the IPV immunization coverage in this study was also lower than full childhood immunization coverage in Ethiopia (65%), which was reported by a systematic review and meta-analysis of observational studies [18]. The discrepancy could be due to the fact that the coverage of this study was based on the interview responses of the mothers of children, while the overall coverage was based on observational findings.

According to the spatial analysis of this study, there were variations in the pattern and predicted rates of vaccination for the inactivated polio vaccine across Ethiopia. Analogous findings were demonstrated by earlier studies [19, 20]. On that account, the rates of inactivated poliovirus vaccine immunization were observed to be greater in Addis Ababa, Tigiray, some parts of Amahara, Dire Dawa and Beninshangul Gumuz provinces and lower in the Somali, Afar, and SNNPR provinces of Ethiopia. The observed low uptake of the inactivated poliovirus vaccine in Somali, Afar, and SNNPR may be attributed to a number of factors, including inadequate health and transportation infrastructure, a lack of knowledge regarding childhood immunization, restricted access to the vaccine, and misinformation about vaccinations among the pastoralist and nomadic populations [21–23], Getting health care services, especially immunizations, is difficult for the seasonal migrants who comprise the pastoralist and nomadic populations [24].

The multilevel mixed effect logistic regression analysis of this study found that two individual level (ANC visit and place of delivery) and two community level factors (residence and region) were significantly associated with childhood inactivated poliovirus vaccine immunization. This is in agreement with findings of studies on other vaccines [25, 26].

The odds of IPV vaccination were higher among children born to mothers who had ANC visits compared to children born to mothers who had no visits. This is in line with several previous studies on IPV and other vaccines [25–28]. This could be as a result of the counselling and instruction mothers received about the value of postnatal visits and activities during ANC visits [28]. The ANC visits offer women an opportunity to learn about immunizations, and mother education plays a significant role in ensuring that children receive the necessary vaccinations [26]. Hence, public health services should continue to prioritize ANC visits and address barriers to education and awareness in order to raise childhood immunization rates.

In addition, the odds of IPV immunization were higher among children born at health facility compared to those born at home. The same was reported from a study conducted in Ethiopia and sub-Saharan Africa to ascertain the connection between infant vaccination and health facility-based delivery [29–32]. In addition to the potential to lower maternal mortality and early newborn deaths associated with delivery-related events, health facility delivery encourages new mothers to vaccinate their newborns after delivery [30]. It appears that mothers who deliver at the facility interact with the healthcare system in a way that encourages their children to seek care in the coming years.

The place of residency was significantly associated with IPV immunization. When compared to children who live in rural areas of Ethiopia, the odds of IPV immunization were higher among children who live in urban areas. This finding agrees with previous studies on childhood immunization [33–37]. A study conducted to assess the role of place of residency in childhood immunization coverage found that there was higher childhood vaccination in urban areas compared to rural areas [37]. Beyond the urban-rural disparities, the study found that vaccination rates were higher in formal urban areas than in slum areas [37]. The rationale behind this finding could be that parents in remote areas lack awareness about child immunization, have poor levels of education, a low wealth index, and live a long way from health facilities [38].

Moreover, region was another community level factor associated with IPV immunization. Previous study on other vaccines have also reported similar finding [31, 37]. Taking Somali as reference, the odds of IPV immunization were higher in Tigray, Amhara, Addis Ababa and Benishangul regions, respectively. A sparse population density and poor health infrastructure have been suggested as obstacles to the provision of immunization services in the Somali region [39, 40]. Furthermore, the Somali region is mostly home to a pastoralist nomadic community [40].

The use of multilevel mixed-effect logistic regression to determine two-level factors, which was not possible by using conventional logistic regression, was the study’s strength. Nonetheless, since the study was based on secondary data, it was impossible to include other variables that could have an impact on the outcome variable. Another limitation of this study was using data from a mini-demographic and health survey, which was conducted on only half of the clusters usually surveyed in the full demographic survey in Ethiopia. Moreover, due to the cross-sectional nature of the data used for the study, the association of individual and community-level factors with IPV immunization should not be regarded as casual associations.

## Conclusion

The distribution of inactivated poliovirus immunization was substantially variable spatially across Ethiopia. Only about half of the children aged twelve to thirty-five months received the inactivated poliovirus vaccine in the country. The factors, both at the individual and community level, were significantly associated with inactivated poliovirus immunization. Therefore, policies and strategies could benefit from considering antenatal care follow-up, place of delivery, place of residence, and region while implementing inactivated poliovirus vaccine immunization.

## Abbreviations

ANC: Antenatal Care
AOR: Adjusted Odds Ratio
CI: Confidence interval
DHS: Demographic and Health Survey data
EMDHS: Ethiopian Mini Demographic and Health Survey
ICC: Intra-cluster Correlation Coefficient
IPV: Inactivated Poliovirus Vaccine
LL: Log likelihood
LLR: Log Likelihood Ratio
MOR: Median Odds Ratio
PCV: Proportional Change in Variation
SNNPR: Southern Nations Nationalities and People’s Region
